# Chimeric Bivalent Virus-Like Particle Vaccine for H5N1 HPAI and ND Confers Protection against a Lethal Challenge in Chickens and Allows a Strategy of Differentiating Infected from Vaccinated Animals (DIVA)

**DOI:** 10.1371/journal.pone.0162946

**Published:** 2016-09-14

**Authors:** Jin-Yong Noh, Jae-Keun Park, Dong-Hun Lee, Seong-Su Yuk, Jung-Hoon Kwon, Sang-Won Lee, Joong-Bok Lee, Seung-Yong Park, In-Soo Choi, Chang-Seon Song

**Affiliations:** Avian Disease Laboratory, College of Veterinary Medicine, Konkuk University, Seoul, Korea; Sun Yat-Sen University, CHINA

## Abstract

Highly pathogenic avian influenza (HPAI) and Newcastle disease (ND) are considered as the most devastating poultry infections, owing to their worldwide distribution and economical threat. Vaccines have been widely used to control these diseases in the poultry industry in endemic countries. However, vaccination policy without differentiating infected animals from vaccinated animals (DIVA) makes the virus surveillance difficult. In this study, we developed a bivalent virus-like particle (VLP) vaccine that is composed of the hemagglutinin (HA) and matrix 1 (M1) proteins of the H5N1 HPAI virus (HPAIV) and a chimeric protein containing the ectodomain of the ND virus (NDV) fusion (F) protein fused with the cytoplasmic and transmembrane domains of the HPAIV HA protein. A single immunization of chickens with the chimeric VLP vaccine induced high levels of hemagglutination inhibition (HI) antibody titers against H5N1 HPAI virus and anti-NDV antibody detected in ELISA and protected chickens against subsequent lethal HPAIV and NDV infections. Furthermore, we could easily perform DIVA test using the commercial NP-cELISA tests against HPAIV and HI assay against NDV. These results strongly suggest that utilization of chimeric VLP vaccine in poultry species would be a promising strategy for the better control of HPAI and ND simultaneously.

## Introduction

Highly pathogenic avian influenza (HPAI) and Newcastle disease (ND) are devastating diseases in poultry with mortality rates of up to 100% and are classified as OIE-listed diseases [1, 2]. The Asian origin A/goose/Guangdong/1/1996 (H5N1) lineage of HPAI virus (HPAIV) has spread across four continents and has become endemic in many countries after it was first detected in China, 1996 [3, 4]. In addition, total 449 cases of H5N1 HPAI have caused fatalities in humans until 13, November, 2015 in WHO report; thus, the H5N1 HPAIV has been considered as a pandemic virus [5]. ND is caused by the virulent avian paramyxovirus serotype 1 (APMV-1) that is transmissible to poultry and to over 240 other species of birds [6]. Mortality from infection with virulent ND virus (NDV) can reach 100% in poultry flocks of fully susceptible chickens [7].

HPAIV and NDV have become endemic in the poultry of many countries in Southeast Asia and vaccination for both diseases are commonly used in these countries [8, 9]. In these countries, differentiating infected animals from vaccinated animals (DIVA) is important to make virus surveillance easier. However, conventional HPAI and ND vaccines have been primarily with killed whole virus-adjuvanted vaccines and live attenuated vaccines, respectively, DIVA cannot be easily carried out [10, 11]. Therefore, diverse different DIVA strategies have been proposed using appropriate vaccines and companion serologic tests for successful discriminating between naturally infected and vaccinated animals. Particularly, since the subunit vaccine contains only immunogenic proteins, it allows DIVA with serologic test against the viral protein that is not incorporated into vaccine [12].

Virus-like particles (VLPs), which morphologically resemble authentic viral structures but are not infectious, have been suggested as an alternative candidate for conventional vaccines [13]. VLP vaccines produced by using the most prominent immunogenic proteins of viruses have been shown to elicit high protective efficacies against various viral pathogens [14–16]. These vaccines provide numerous advantages over conventional vaccines, including safety, immunogenicity, and multivalency. Currently, several different VLP-based multivalent vaccine platforms have demonstrated that chimeric VLP vaccines induce a protective efficacy against multiple viral pathogens simultaneously [17, 18]. In addition, since VLPs only include immunogenic and core proteins of viruses, it allowed DIVA strategy by using classical diagnostic methods [19].

The influenza virus HA protein, as a major surface glycoprotein, is considered the most important antigen and can elicit neutralizing antibodies [20]. In case of NDV, antibodies to the F protein appear to be predominantly necessary to prevent infection and spread of the virus [21]. The AIV M1 protein can accommodate various viral surface proteins, thus it has been suggested as a universal core protein of VLP to incorporate foreign proteins [22–24].

In this study, we developed chimeric VLPs in insect cell lines expressing HA and M1 proteins of the HPAIV and the F/HA protein, a chimeric protein containing the ectodomain of the NDV F protein fused with transmembrane and cytoplasmic domains (TM/CT) of the HPAIV HA protein. We evaluated its immunogenicity and protective efficacy in specific-pathogen-free (SPF) chickens and assessed DIVA-ability of VLP vaccines by using nucleoprotein (NP) coated ELISA test for HPAIV and Hemagglutination Inhibition (HI) test for NDV.

## Materials and Methods

### Ethics statement

All animal procedures carried out in this study (permit number: KU14008) were reviewed, approved, and supervised by the Institutional Animal Care and Use Committee (IACUC) of Konkuk University.

### Cells and viruses

Sf9 insect cells (Gibco, USA) were maintained in suspension in SF-900 III SFM medium (Gibco, USA) at 27°C. The HPAI A/chicken/Korea/ES/2003 (H5N1) ES03 virus and velogenic ND virus KR-005/00 were kindly provided by the Animal and Plant Quarantine Agency, Korea. Viruses were propagated in 9-11-day-old SPF chicken embryonated eggs and used for further experiment.

### Cloning of HA, M1, and F/HA chimeric genes

**Fig 1:**
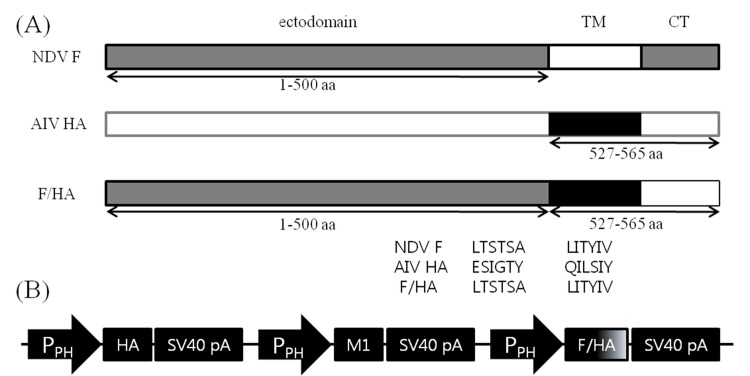
Baculovirus construct for production of chimeric virus-like particles. (A) construction of a F/HA fusion gene containing ectodomain of the NDV KR-005/00 strain F gene and TM/CT domain of the H5N1 avian influenza virus ES03 strain HA gene. The schematic diagram shows the location of the ectodomain, transmembrane (TM) domain, and cytoplasmic tail (CT) domain of the F and HA genes and their junction. The TM/CT domain of the HA gene was fused to the carboxyl terminal of the F gene to form the full-length F/HA fusion gene; (B) HA, M1, and chimeric F/HA genes with its own polyhedrin promoter (Pph) and transcription termination sequences are indicated.

HA (Genbank AY676035) and M1 (Genbank AY676047) genes of A/chicken/Korea/ES/2003 and the F gene (Genbank AV630423) of KR-005/00 were extracted from virus infected allantoic fluid and amplified by two-step reverse transcription-polymerase chain reaction (RT-PCR; RNeasy® Mini Kit, Omniscript™ RT Kit, Qiagen, Germany). For cDNA synthesis, Uni12 primer was used for HA and M1 genes and a 5′-ATGGGCTCCAAACTTTC-3′ primer for F gene, as previously described [25, 26]. To enhance the interaction between the ectodomain of the NDV F and the AIV M1 proteins in particle assembly, we constructed a chimeric F/HA protein. The ectodomain of the F gene was generated by PCR and the codon optimized HA TM/CT domain was synthesized by Bioneer, Korea. The two products were mixed equally and F/HA chimeric gene was constructed by overlap PCR using primers EcoRI-F-1 and HindIII-HA-TM/CT-2 (Fig 1A and Table 1). Sequences of primers used to amplify each segment are shown in Table 1. Amplified genes were cloned into the TA vector (RBC Bioscience, Taiwan) and sequenced. The three resulting plasmid vectors were designated as vHA, vM1, and vF/HA.

**Table 1:** Primer sets used for RT-PCR amplification of genes of HPAIV ES03 and NDV KR-005/00.

Target gene	Primer (5’-3’)	Expected size(bp)
HA	BamHI-HA-1:GGA TCC ATG GAG AAA ATA GTG C	1695
	HindIII-HA-2:AAG CTT AGT AGA AAC AAG G	
M1	EcoRI-M1-1:GAA TTC ATG AGT CTT CTA ACC GAG G	759
	HindIII-M1-2:AAG CTT TCA CTT GAA TCG CTG C	
F(TM/CT removed)	EcoRI-F-1:GAA TTC ATG GGC TCC AAA CTT TC	1500
	XhoI-F-2:CTC GAG AGC AGA TGT GCT GGT TAG	
HA TM/CT	XhoI-HA-TM/CT-1:CTC GAG CAG ATC CTG TCC ATC TAC	117
	HindIII-HA-TM/CT-2:AAG CTT TTA GAT GCA GAT ACG GCA C	

### Construction of expression vector

The plasmid vector vHA was digested with BamHI/HindIII and vectors vM1 and vF/HA were digested with EcoRI/HindIII. The resulting fragments were ligated into a pFastBac^TM^1 bacmid transfer vector (Invitrogen, USA) that had been digested with the same enzymes, resulting in plasmid vectors pHA, pM1, and pF/HA. Plasmid vectors pHA and pF/HA were digested by SnaBI/HpaI, and digested fragments were ligated into SnaBI and HpaI sites of pM1, respectively, to generate pHAM1F/HA. The resulting plasmid containing the influenza virus HA, M1, and chimeric F/HA genes with its own polyhedrin promoter and transcription termination sequences was confirmed in its entirety to ensure that no additional changes were introduced (Fig 1B).

### Transfection and generation of recombinant baculovirus

pHAM1F/HA vector was transformed into DH10Bac™ competent cells (Invitrogen) to generate a recombinant bacmid according to manufacturer’s instructions. The recombinant bacmid was isolated by PureLink™ HiPure Plasmid Miniprep Kit (Invitrogen) and transfected into Sf9 cells by using the Cellfectin® II Reagent (Invitrogen). At 72 h post-transfection, budded recombinant baculoviruses (rBVs) released into the media were harvested and inoculated into Sf9 cells to generate a high-titer rBV stock. Titration of baculovirus stock was performed by plaque assay according to user manufacturer’s recommendation (Invitrogen).

### Production of chimeric VLP

For chimeric VLP production, Sf9 cells at a concentration of 2 × 10^6^ cells/ml were infected with rBVs expressing HA, M1 and F/HA proteins at a multiplicity of infection (MOI) of 5 for 3 days. The cultured media were collected and centrifuged at 2000 × g for 10 min to remove cell debris.

VLPs in the supernatants were pelleted by ultracentrifugation at 18,000 rpm for 1.5 h at 4°C by using a 19 Ti rotor (Beckman, USA) to concentrate, resuspended in phosphate-buffered saline (PBS), and purified by sucrose gradient using 20% and 50% sucrose solutions (g/ml) at 35,000 rpm for 1.5 h at 4°C by using a SW 41 Ti rotor (Beckman). The VLP bands were collected and analyzed by Western blots using mouse anti-H5 monoclonal antibody (#12E73B, Bionote, Korea) and mouse anti-Kr-005/00 F monoclonal antibody (#10C66, Median Diagnostics, Korea). HRP-conjugated goat anti-mouse IgG antibody (Bethyl, USA) was used as the secondary antibody for detection. The total protein concentrations of the VLP vaccine preparations were determined by using Bradford protein assay (Thermo Scientific, USA).

### Electron microscopy analysis

Chimeric VLPs were absorbed by flotation onto a freshly discharged 400-mesh carbon-coated copper grid. The grids were rinsed, negatively stained with 2% uranyl acetate, and dried by aspiration. The VLPs were visualized using a transmission electron microscope (BIO-TEM, Tecnai G2 Spirit, installed at Korea Basic Science Institute) operating at 120 kV at 100,000x magnification.

For immunoelectron microscopy of chimeric VLPs, samples were absorbed and blocked with 1% BSA in PBS. VLPs were probed with the mixture of 1:10 diluted mouse anti-H5 monoclonal antibody (#12E73B, Bionote, Korea) and 1:2 diluted chicken antiserum against NDV KR005. Secondary antibodies were mixture of 1:10 diluted goat anti-mouse IgG labeled with 10nm gold particles (G7652, Sigma Aldrich, U.S.) and 1:2 diluted rabbit anti-chicken IgG labeled with 25nm gold particles (25593, Electron Microscopy Science, U.S.).

### Preparation of VLP vaccine

VLP vaccines were prepared by emulsifying purified VLP with Montanide ISA 70 adjuvant (SEPPIC, France) at a ratio of 30:70 (w⁄w) to obtain final vaccine concentrations of 10 μg/chicken and 2 μg/chicken. Mock vaccine was prepared with PBS by using same method as with VLP vaccines.

### Animals and experimental design

Thirty 6-week-old SPF chickens were divided into 3 groups (n = 10) and housed in different isolators under biosafety level 3 conditions. Two groups of chickens were immunized intramuscularly with 0.5 ml of the 10 μg/chicken or 2 μg/chicken HAM1F/HA VLP vaccine, and the challenge control group was vaccinated with mock vaccine.

Three weeks after a single immunization, chickens were challenged intranasally with 100 μl of 10^6.0^EID_50_/dose of wild-type ES03 HPAIV. To evaluate protective efficacy of the HAM1F/HA VLP vaccine, mortality and clinical signs were observed twice daily for 14 days post-challenge (dpc) without analgesic or anaesthetics and chickens were euthanized by cervical dislocation when they were reached a predetermined humane endpoint. A humane endpoint was determined when chickens showed neurological signs like head tilt and ataxia, or sever edema and cyanosis of wattles, comb, periorbital tissue, or markedly reduced activity. In addition, virus shedding was quantified at 2, 3, 5, and 7 dpc by real-time RT-PCR (rRT-PCR) in oropharyngeal and cloacal swabs to determine the level of shedding. In detail, swab samples were collected and suspended in 1 ml of PBS supplemented with gentamycin (400μ/ml) and stored at -70°C before further process. Of this suspension, 150 μl was used for RNA extraction with Viral Gene-spin™ (Intron, Korea) according to the manufacturer’s instructions. The total amount of RNA was quantified by the cycle threshold (Ct) method by using M gene-based rRT-PCR, as previously described [27].

Similarly, thirty 6-week-old SPF chickens were divided into 3 groups (n = 10) and housed in different isolators under biosafety level 2 conditions. Two groups of chickens were immunized intramuscularly with 0.5 ml of the 10 μg/chicken or 2 μg/chicken chimeric VLP vaccine. The challenge control group was vaccinated with mock vaccine.

Three weeks after a single vaccination, all groups of chickens were challenged intramuscularly with 1 ml of 10^5.5^EID_50_/ml of the velogenic NDV Kr-005/00. To assess the protective efficacy, mortality and clinical symptoms were observed twice daily for 14 dpc and euthanized when chickens were reached same criteria with HPAI experiment.

### Serology

In order to determine the efficacy of VLP vaccine, serum samples were collected prior to vaccination and at 3 weeks after vaccination for the HI test for AIV and ELISA for NDV. For AIV, collected serum samples were tested for the presence of HI antibodies according to the OIE standard method using formalin-inactivated homologous AIV. For NDV, sera were analyzed using a commercially available ELISA kit (Median Diagnostics) pre-coated with the NDV La Sota strain (Genbank JF950510.1) according to manufacturer’s instructions.

### DIVA

For serological differentiation of chimeric VLP-vaccinated chickens from vaccinated and then infected chickens, commercial competitive NP-ELISA was used for AIV according to manufacturer’s instructions (BioNote) and the HI test for NDV La Sota strain was done according to OIE standard method. Because the chimeric VLPs developed in this study do not contain AIV nucleoproteins (NP) and NDV hemagglutinin-neuraminidase (HN) proteins, levels of anti-NP antibodies for AIV and HI antibodies for NDV were expected to increase only after virus infection. Serum samples were collected at 0 dpc, which is 3 weeks post-vaccination, and at 14 dpc from all surviving chickens challenged with lethal HPAIV or NDV.

### Statistical Analysis

Analysis of variance (ANOVA) along with a Tukey–Kramer post-hoc test was performed for comparison of serum antibody titers between groups. Results with P values < 0.05 were considered statistically significant.

## Results

### Chimeric VLP expression

**Fig 2:**
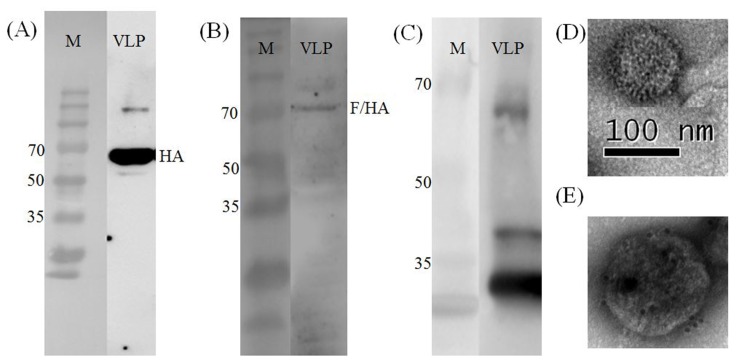
**Analysis of concentrated chimeric VLP:** (A) Western blot identification of the HA protein using anti-H5 monoclonal antibody. M, standard molecular size marker (EBM-1032, Elpisbio), HA, chimeric VLPs; (B) Western blot identification of the F/HA chimeric protein using anti-NDV F monoclonal antibody; (C) Western blot identification of the M1 protein using anti-H5N1 anti-serum; (D) TEM identification of chimeric VLP with scale bar; (E) immunogold-labelled chimeric VLP: VLP were probed with anti-H5 monoclonal antibody, counterstained with 10nm gold spheres coupled to anti-mouse IgG and anti-NDV anti-serum, counterstained with 25nm gold spheres coupled to anti-chicken IgG.

Chimeric VLP containing HA and M1 proteins of A/Chicken/Korea/ES/2003 (H5N1) and the F/HA chimeric protein of KR-005/00 and A/Chicken/Korea/ES/2003 (H5N1) was successfully produced and secreted from Sf9 cells. The HA titer of VLP from culture supernatant was 256 HAU/50ul and VLPs were purified from the culture supernatant. HA, F/HA and M1 proteins were detected by Western blot analysis (Fig 2A and 2B) and VLPs were detected by transmission electron microscopy (TEM) (Fig 2C).

### Immune response

**Fig 3:**
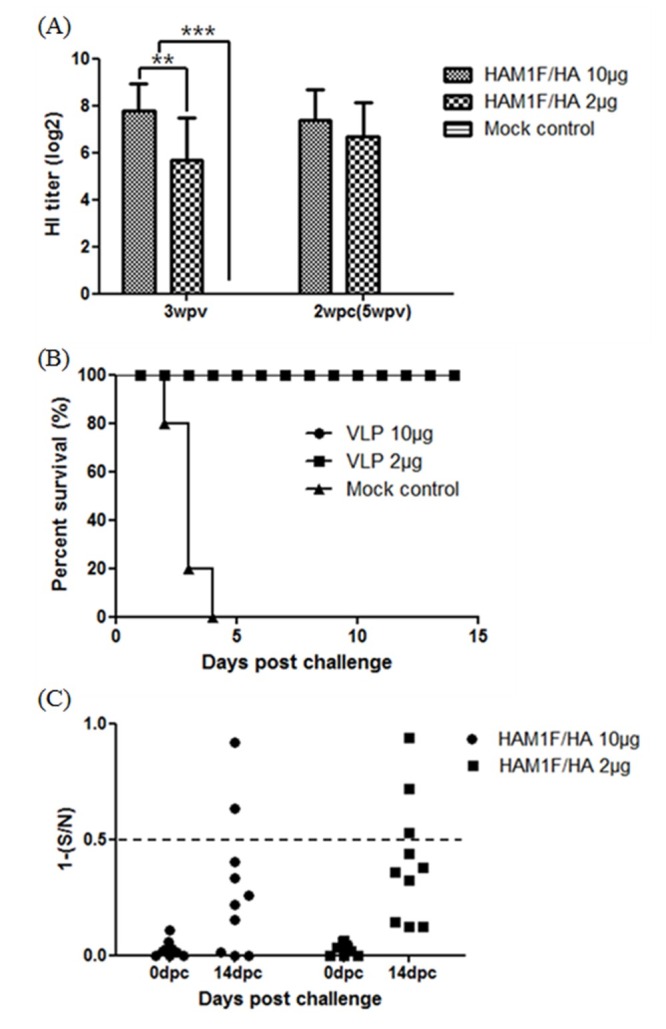
HPAIV Experiments in SPF chickens. (A) HI titers after three weeks post vaccination and two weeks after HPAIV challenge: serum samples were analyzed with the standard HI test for the presence of HI antibodies against ES03 H5 AIV. Error bars represent standard deviations. ***, P < 0.001, **, P < 0.01, (B) Survival curve after lethal HPAIV challenge. Three weeks after immunization, SPF chickens were intranasally infected with a lethal dose of ES03 HPAI virus. The chickens were observed daily for a period of 14 days for survival. (C) Differentiation of vaccinated chickens from vaccinated and infected chickens. Serum samples were taken before challenge (3 weeks post-vaccination) and 14 days post challenge. NP antibody levels were tested with a commercially available NP-cELISA kit. Each dot represents the NP-specific antibody value of each chicken.

For HPAIV, at three weeks after a single vaccination, all VLP-vaccinated groups showed significantly increased HI antibody titers (GMT = 222.8) to homologous H5 HPAIV compared to the mock vaccinated group (GMT = 0) (P < 0.001). Additionally, the 10 μg/chicken-vaccinated group showed higher HI antibody titers than the 2 μg/chicken-vaccinated group (GMT = 52.0) in dose-dependent manner (P < 0.01) (Fig 3A).

**Fig 4:**
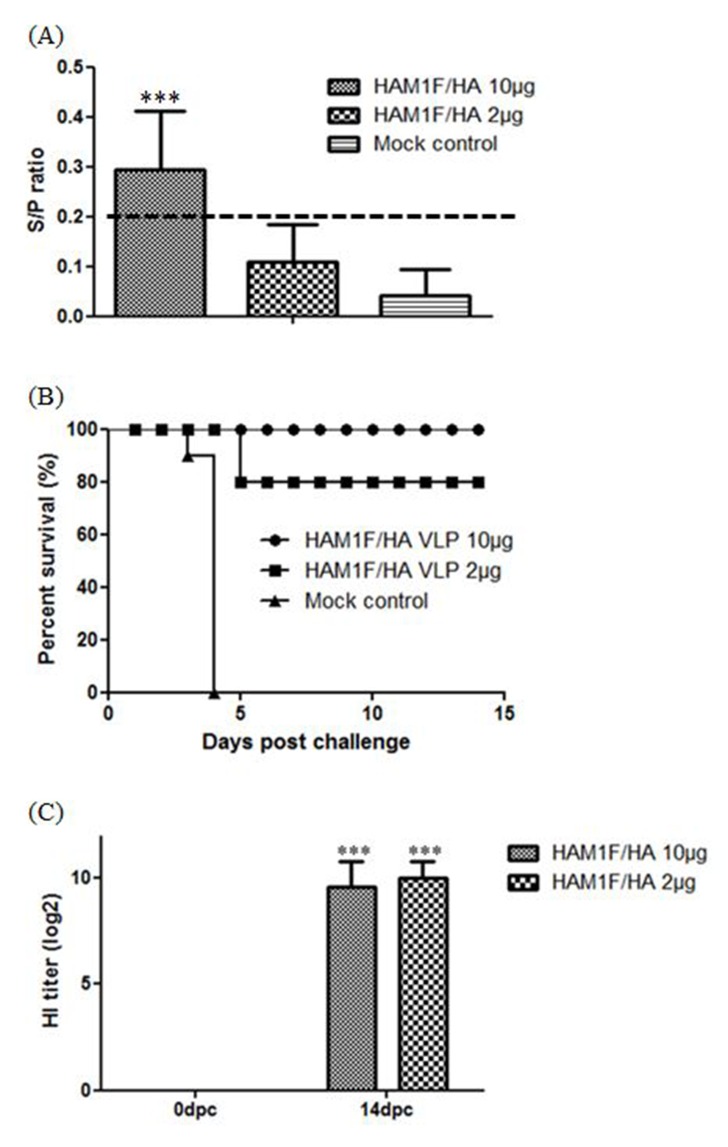
NDV Experiments in SPF chickens. (A) Mean levels of antibody against NDV in serum. Levels of antibody against NDV in serum were determined using a commercially available ELISA kit after three weeks post vaccination. The cut-off value was presented as dot line. Statistical significance was determined by ANOVA with the Tukey-Kramer post hoc test. ***, P < 0.001; **, P < 0.01 (B) Survival curve after lethal NDV challenge. Three weeks after immunization, SPF chickens were intramuscularly infected with a lethal dose of KR-005/00 strain NDV. The chickens were observed daily for a period of 14 days for survival. (C) HI titers after two weeks post challenge for DIVA: Serum samples were analyzed with the standard HI test for the presence of HI antibodies against the La Sota NDV strain. Error bars represent standard deviations. ***, P < 0.001,

For NDV, at three weeks after a single vaccination, the 10 μg/chicken-vaccinated group showed significantly increased antibody titers in ELISA compared to the 2 μg/chicken-vaccinated and mock vaccinated groups (P < 0.001). The 2 μg/chicken-vaccinated group showed higher antibody titers than the mock vaccinated group, but it was observed lower than positive cutoff (0.2) (Fig 4A).

### Protection and virus shedding

To examine the protective efficacy of the vaccine against HPAIV infection, chickens were challenged intranasally with a lethal dose of HPAI ES03 virus at 3 weeks after vaccination. Mock-vaccinated chickens showed 100% mortality within 4 dpc. However, all of the VLP-vaccinated chickens were protected from mortality and presented no clinical signs (Fig 3B). All vaccinated birds showed reduced virus shedding, both in the oropharynx and cloaca, than mock-vaccinated chickens (Tables 2 and 3). In oropharyngeal shedding, only one chicken in the 10 μg/chicken-vaccinated group showed little viral shedding at 3 dpc and no chicken in the 2 μg/chicken-vaccinated group showed virus shedding throughout the experiment period. In cloacal shedding, all vaccinated chickens shedded the virus at 3 dpc, but a relatively low titer of the virus was detected than mock-vaccinated chickens; none of the chickens shed the virus after 5 dpc in oropharynx and cloaca.

**Table 2:** Challenge HPAI virus shedding from oropharyngeal swab samples.

Days post-challenge	HAM1F/HA 10 μg		HAM1F/HA 2 μg		Mock vaccinated	
	No. of positive/total	Avg. Ct	No. of positive/total	Avg. Ct	No. of positive/total	Avg. Ct
2	0/10	-	0/10	-	3/8	30.72
3	1/10	33.98	0/10	-	2/2	30.24
5	0/10	-	0/10	-	N.A.	
7	0/10	-	0/10	-	N.A.	

N.A., Not applicable

**Table 3:** Challenge HPAI virus shedding from cloacal swab samples.

Days post-challenge	HAM1F/HA 10 μg		HAM1F/HA 2 μg		Mock vaccinated	
	No. of positive/total	Avg. Ct	No. of positive/total	Avg. Ct	No. of positive/total	Avg. Ct
2	8/10	33.05	7/10	32.78	5/8	31.33
3	10/10	30.99	10/10	30.13	1/2	27.28
5	0/10	-	0/10	-	N.A.	
7	0/10	-	0/10	-	N.A.	

N.A., Not applicable

For NDV, chickens were intramuscularly infected with a lethal dose of NDV KR-005/00 at 3 weeks after vaccination. Vaccination protected chickens in a dose-dependent manner. 10 μg/chicken-vaccinated group was fully protected from mortality and no clinical signs were observed. Two chickens of the 2 μg/chicken-vaccinated group died 5 days after challenge, but the surviving chickens presented no clinical signs. Mock-vaccinated chickens showed 100% mortality within 4 days after challenge (Fig 4B).

### DIVA

For differentiating vaccinated chickens from vaccinated and infected chickens, sera were collected at 0 and 14 dpc for avian influenza NP-cELISA and HI tests against NDV. For AIV, all VLP-vaccinated groups showed HI antibody titers to homologous H5 HPAIV but were negative for NP-cELISA before challenge. In contrast to pre-challenge sera, 2 of 10 and 3 of 10 samples of post-challenge sera of 10 μg/chicken-vaccinated and 2 μg/chicken-vaccinated birds were positive (Fig 3C).

For NDV, pre-challenge sera from VLP-vaccinated chickens did not show any increase in HI titers. In contrast to pre-challenge sera, post-challenge sera of all VLP-vaccinated chickens showed significantly higher HI antibody titers (GMT = 776.0, 1024.0 repectively), indicating that VLP vaccine with companion serologic test could accomplish successful DIVA strategy (Fig 4C).

## Discussion

In this study, a chimeric VLP containing the influenza HA, M1, and chimeric F/HA proteins was successfully produced. A single vaccination with the chimeric VLP vaccine induced high levels of effective antibodies against both HPAIV and NDV and protected SPF chickens against subsequent lethal infection.

Vaccination of poultry with effective bivalent vaccines against HPAI and ND has potential advantages because a single immunization could protect chickens against both devastating diseases and reduce vaccine costs and labor. Previously, several bivalent vaccines against HPAI and ND viruses have been produced in forms of live attenuated vector vaccines, inactivated vaccines, and VLP vaccines [28–31]. These bivalent vaccines provided efficient clinical protection against both HPAI and ND, but DIVA approach has been successfully applied to AIV, and not been proposed to NDV. However, with chimeric VLP vaccine produced in this study, DIVA tests as well as protection for both HPAIV and NDV infections were successfully performed by using commercial influenza NP-cELISA kits and anti-NDV HI tests. Because the use of influenza NP-cELISA and the HI test for NDV are one of the most common and useful methods for confirming virus infection and subsequent antibody production [32, 33], DIVA based on these tests with VLP vaccine is advantageous in that no additional serological methods will need to be developed.

Interestingly, 2 μg/chicken VLP-vaccinated group showed low S/P ratio in ELISA after 3 weeks post-vaccination for NDV. Additionally, two chickens were dead in 2 μg/chicken VLP-vaccinated group after virulent NDV challenge. However, 10 μg/chicken VLP-vaccinated group showed high S/P ratio and none of the chickens were dead, it is assumed that 10 μg/chicken dose is required for efficient protection for NDV. There are some possibility that F/HA protein was relatively low expressed than HA protein according to Western blot and TEM picture of Fig 2.

In case of HPAIV, all vaccinated chickens showed some levels of virus shedding from cloaca. However, serological tests after HPAIV challenge revealed that not all the vaccinated chickens showed detectable seroconversions against the AIV NP protein and most of the chickens showed no increase in HI titers. These results are in common with some previous studies that no seroconversion was shown after HPAIV infection in vaccinated chickens [34, 35]. This is possibly due to suppressed viral replication which means that virus could not reach sufficient titer to induce immune response against HPAIV. This could confirm that chimeric HAM1F/HA VLP vaccines were well developed and demonstrated complete protection against the virus challenge. However, lack of seroconversion in some chickens could be a potential issue for implementing DIVA strategies because it is hard to detect virus infection by monitoring antibody levels of individual birds. Therefore, for successful DIVA strategies, virus surveillance needs to be based on flock analysis and the use of unvaccinated sentinel birds is recommended.

In conclusion, chimeric VLPs expressed by baculovirus system provide complete protection against HPAIV and NDV in SPF chickens and enable DIVA strategies for both diseases by using conventional serological methods. It is expected that similar approaches will expand the utility of chimeric VLPs in preventing multiple viral diseases by a single vaccination with implementation of DIVA strategies.
